# Effect of Cellulose Nanofibrils on the Properties of Jatropha Oil-Based Waterborne Polyurethane Nanocomposite Film

**DOI:** 10.3390/polym13091460

**Published:** 2021-04-30

**Authors:** Mohamad Ridzuan Amri, Chuah Teong Guan, Syeed Saifulazry Osman Al-Edrus, Faizah Md Yasin, Siti Fatahiyah Mohamad

**Affiliations:** 1 Institute of Tropical Forestry and Forest Product, Universiti Putra Malaysia, Serdang 43400, Selangor, Malaysia; mridzuan.work@gmail.com; 2 Department of Chemical and Environmental Engineering, Faculty of Engineering, Universiti Putra Malaysia, Serdang 43400, Selangor, Malaysia; fmy@upm.edu.my; 3 Institute of Advance Technology, Universiti Putra Malaysia, Serdang 43400, Selangor, Malaysia; 4 Radiation Processing and Technology Division, Malaysia Nuclear Agency, Bangi 43000, Selangor, Malaysia; fatahiyah@nuclearmalaysia.gov.my

**Keywords:** nanocellulose, bio-based polyol, biopolymer, bio-based film, biocomposite

## Abstract

The objective of this work was to study the influence of cellulose nanofibrils (CNF) on the physical, mechanical, and thermal properties of Jatropha oil-based waterborne polyurethane (WBPU) nanocomposite films. The polyol to produce polyurethane was synthesized from crude Jatropha oil through epoxidation and ring-opening method. The chain extender, 1,6-hexanediol, was used to improve film elasticity by 0.1, 0.25, and 0.5 wt.% of CNF loading was incorporated to enhance film performance. Mechanical performance was studied using a universal test machine as specified in ASTM D638-03 Type V and was achieved by 0.18 MPa at 0.5 wt.% of CNF. Thermal gravimetric analysis (TGA) was performed to measure the temperature of degradation and the chemical crosslinking and film morphology were studied using Fourier-transform infrared spectroscopy (FTIR) and field emission scanning electron microscopy (FESEM). The results showed that when the CNF was incorporated, it was found to enhance the nanocomposite film, in particular its mechanical and thermal properties supported by morphology. Nanocomposite film with 0.5 wt.% of CNF showed the highest improvement in terms of tensile strength, Young’s modulus, and thermal degradation. Although the contact angle decreases as the CNF content increases, the effect on the water absorption of the film was found to be relatively small (<3.5%). The difference between the neat WPBU and the highest CNF loading film was not more than 1%, even after 5 days of being immersed in water.

## 1. Introduction

In recent years, bio-based polyurethane has become a consumer preference and of interest to researchers. Two types of polyurethane (PU) are available, i.e., solvent and water-based polyurethane. Waterborne polyurethane is one of the environmentally friendly materials in the field of surface coatings, and water-based or waterborne polyurethane is not aqueous, but rather a well-dispersed mixture stabilized by electrostatic repulsive force [1]. The focus of PU in industry and academic research has shifted towards waterborne PU dispersion (WBPU) due to its replacement of volatile chemicals by water as a solvent in the production of PU. In addition, WBPU also offers several advantages, such as high flexibility at low temperatures, pollution-free, non-inflammable, good applicability, and non-toxic [2,3]. WBPU can be considered as a ‘green’ coating material compared to conventional PUs since it does not release any volatile organic compounds during the curing process. Jatropha oil consists of oleic acid (18:1), linoleic acid (18:2), palmitoleic acid (16:1), and linoleic acid (18:3) [4]. The presence of unsaturated fatty acids makes Jatropha oil a potential material for the production of polyols and the production of polyurethane. The extraction of polyols from Jatropha oil involves two consecutive steps: epoxidation and oxirane ring-opening. During epoxidation, the unsaturated chain of Jatropha oil is used to form a ring-shaped epoxy group. Oxygen in the epoxy group results from the decomposition reaction of hydrogen peroxide with the fatty acid of Jatropha oil. Interestingly, in the oxirane ring-opening phase, which is the second step in the extraction of polyol, hydroxylation occurs, where the ring shape of the epoxy group is broken and forms a hydroxyl group due to the reaction with methanol. Epoxidation and oxirane ring-opening are extremely sensitive to any changes in temperature, therefore precautions are needed to avoid overheating of the reaction mixture [5]. Polyurethane is produced by the reaction between polyol and isocyanate, which refers to soft and hard segments, respectively. Jatropha oil-based polyol is then reacted with isophorone diisocyanate (IPDI). The addition of dimethylol propionic acid (DMPA) was followed by dispersion at 1200 rpm with essential deionized water. DMPA acts as an internal emulsifier to build a hydrophilic group into the PU backbone. The resulting mixture is a waterborne polyurethane [6].

There are a number of nanofillers that can be used to produce nanocomposites. These nanofillers are carbon nanotube [7,8,9], nano-silica [10,11], graphite and its derivatives [12,13,14], nanosilver [15,16,17], and nanoclay [18]. However, these nanofillers are not preferred due to their tendency to sediment easily during the mixing and curing process of the nanocomposite. Nanocellulose, i.e., cellulose nanofibrils (CNF), is hydrophilic in nature, unlike the nanofillers mentioned above, and can be homogeneously mixed with any water solvent-based polymer. Nanocellulose reinforcement makes WBPU nanocomposites ideal for ‘green’ coating materials. Most of the previous research has been done using CNF as a filler and it has been reported that it increases the strength of the composite film and shifts the temperature of degradation to a higher temperature [19,20,21,22,23]. However, the hydrophilicity characteristics of the cellulosic material may have an impact on the composite material as it may attract moisture/water to be absorbed [24]. Film performance may affect or deteriorate if the composite contains a large amount of moisture/water. Since the application of polyurethane is significant in the application of the coating, the water/moisture behavior must be minimal or non-existent.

As far as the author is concerned, there is no information or study involving CNF in waterborne Jatropha oil-based polyurethane. A Jatropha oil-based waterborne polyurethane nanocomposite film containing CNF as a filler was therefore prepared and characterized in the present study. The effect of CNF on the physical, mechanical, and thermal properties of this nanocomposite film will be investigated.

## 2. Materials and Methods

### 2.1. Materials

Crude Jatropha oil was supplied by Biofuel Bionas Sdn Bhd, Kuala Lumpur, Malaysia. Formic acid (98%), pyridine (95%), and N-methyl pyrillidone (NMP) (98%) were purchased from Fisher Scientific, Pittsburgh, PA, USA. Triethylamine (TEA) (30%), hydrogen peroxide, dibutyltin dilaurate (DBTL) (98%), methanol (99.8%), phthalic anhydride, dimethylol propionic acid (DMPA), acetone (reagent grade), sodium hydroxide 0.5 N, and magnesium sulfate was purchased from R&M chemicals, Tamil Nadu, India. Isophrene diisocyanate (IPDI) (98%) was purchased from Merck, Germany, and 1–6 Hexanediol (HDO) was purchased from BDH Chemical LTD, Poole, England. Cellulose nanofiber was obtained from the Institute of Tropical Forestry and Forest Product (INTROP) (Serdang, Malaysia). All chemicals were reagent grade and were used as received.

### 2.2. Preparation of Jatropha Oil-Based Polyol (JOL)

Epoxidized Jatropha oil (EJO) was synthesized according to the method reported by Saalah et al., 2014 [6]. Jatropha oil-based polyol (JOL) was poured into a four-neck flask equipped with a mechanical stirrer containing a torque meter, a heater with a temperature sensor, and a dropping funnel. Methanol and water were added to the flask followed by the addition of sulphuric acid. The mixture was then heated to 64 °C before the addition of EJO and the reaction lasted for 30 min. To quench the reaction, sodium bicarbonate was added. The solution was transferred to the separating funnel, cooled to room temperature, and the deposited layer was discarded. Methanol and water were then removed by vacuum distillation at 60 °C for a period of 30 min at 100 rpm. This resulted in a clear golden yellow polyol, which was analyzed for the hydroxyl (OH) number.

### 2.3. Preparation of Jatropha Oil-Based Polyurethane Dispersion

**Figure 1:**
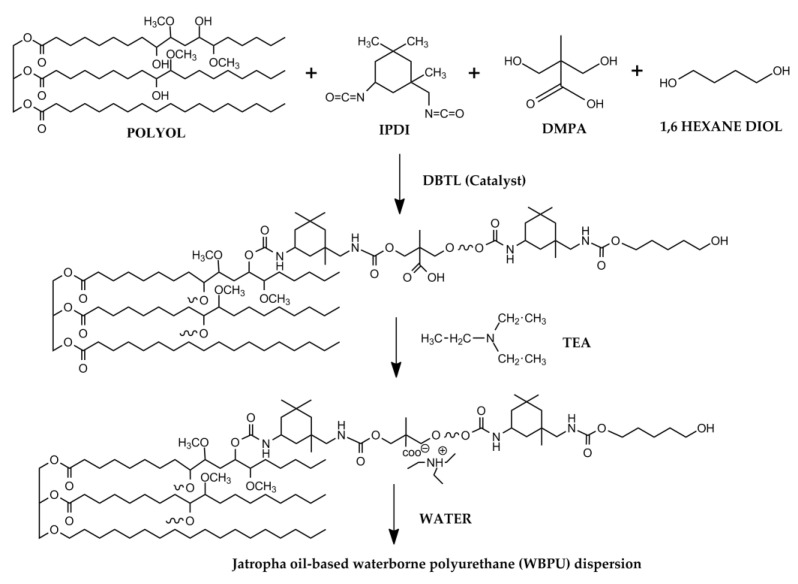
Reaction scheme of WBPU [6,22].

The calculated amount of DMPA (dissolved in NMP) was added into a four-neck flask together with JOL. The four-neck flask was equipped with a mechanical stirrer containing a torque meter, a temperature sensor heater, a nitrogen inlet, and a dropping funnel. The mixture was stirred at 400 rpm for 30 min and heated to 70 °C for a homogeneous mixture. 1 mL of DBTL was added as a catalyst and the reaction was set for 30 min. The IPDI was then added dropwise for 30 min and the agitation increased to 700 rpm. As the IPDI was completed after the addition, the reaction temperature was increased to 80 °C. At the same time, acetone was added batch by batch to control the system’s viscosity. After 2 h of additional reaction time, HDO was added. The reactant was cooled down to 35 °C and TEA was added to neutralize the DMPA followed by dispersion at 1200 rpm with deionized water to produce WBPU with a solid content of 38 wt.%. Acetone was immediately removed using a rotary evaporator and under vacuum conditions. The detailed formulation and reaction scheme of the WBPU is shown in Table 1 and Figure 1.

### 2.4. Preparation of WBPU-CNF Nanocomposite Film

WBPU-CNF nanocomposites films were prepared by the film casting method. The different ratios of the CNF suspension were sonicated for 30 min before the WBPU solution was added. The CNF to WBPU ratio was 0.1, 0.25, and 0.5 wt.%, respectively. The mixture was then poured into the Teflon mold and stored in the desiccator for 7 days and in the vacuum oven for 12 h at 60 °C. The cured film had a thickness of 0.4 mm. The nanocomposite films were labeled as WBPU, WBPU-0.1, WBPU-0.25, and WBPU-0.5 according to their CNF loadings.

### 2.5. Characterisation

#### 2.5.1. Fourier Transform Infrared Spectroscopy (FTIR) Characterisation

The chemical structure of the WBPU and WBPU-CNF was analyzed using FTIR spectra by Perkin-Elmer, Spectrum 2000 series, manufactured in the Beaconsfield, United Kingdom equipped with horizontal germanium attenuated total reflectance (ATR). The spectra were recorded in a range of 4000 to 500 cm^−1^ with a nominal resolution of 4 cm^−1^.

#### 2.5.2. Mechanical Properties Characterisation

The mechanical properties of WBPU and WBPU-CNF film were determined by the INSTRON, 3300 series universal tensile machine, made in the Norwood, MA,, USA, as specified by ASTM D638-03 Type V. The crosshead speed was 10 mm/min, with a load cell of 1 KN. Data from stress and strain measurements were analyzed for Young’s modulus, tensile strength, and elongation at break. The value reported for each sample was from an average of three measurements. The measurement was carried out at room temperature and 50% relative humidity.

#### 2.5.3. Analysis of Thermal Degradation (TGA)

The thermal property analysis of the WBPU and WBPU-CNF films was performed using a TA instrument, Q500 series, manufactured in the New Castle, DE, USA. The films were heated from 25 to 600 °C, at a rate of 10 °C/min, under a nitrogen atmosphere.

#### 2.5.4. Morphology Evaluation

The morphology of the WBPU and WBPU-CNF was analyzed using a field emission scanning electron microscope (FESEM) by Thermo Fisher Scientific, Nova Nanosem 230 series, manufactured in the MA, USA. The samples were sputtered with a layer of gold. The images were then taken up to 50 K magnification with a 5 kV accelerating voltage.

#### 2.5.5. Water Uptake Characterisation

Water uptake measurement is one of the important factors that characterize hydrophilic composite films, especially since CNF is a hydrophilic water-like material. The determination of the water uptake was carried out using the method adopted by Fang et al., 2014 [25]. WBPU and WBPU-CNF composite films were prepared by cutting the film into a 5 × 5 mm sample size with triplicate samples. The cut samples were immersed in deionized water for 5 days. The weight of the specimens were taken after a duration of 2, 6, and 12 h, and followed by every 24 h subsequently. The percentage of water uptake of each specimen was determined by the difference in the sample weight, according to Equation (1) as follows:

Water uptake (%) = [(Wb − Wa)/Wa] × 100(1)

where Wa is the initial weight of the specimen before immersion and Wb is the weight after immersion, respectively.

#### 2.5.6. Water Contact Angle Characterisation

The film behaviour towards water absorption was observed using the water contact angle test as the CNF that is used in the films is hydrophilic in nature. The static contact angle was observed at 25 °C on the surface of the neat WBPU and WBPU-CNF films using the Attension Theta Optical Contact Angle Tester (Biolin Scientific, Manchester, UK). The amount of 3 μL of deionized water was lowered to the surface of the coating using the sessile dropping method. The contact angle readings were taken on the basis of surface interaction within one minute after the water had dropped.

## 3. Results

### 3.1. Fourier Transform Infrared Spectroscopy (FTIR)

**Figure 2:**
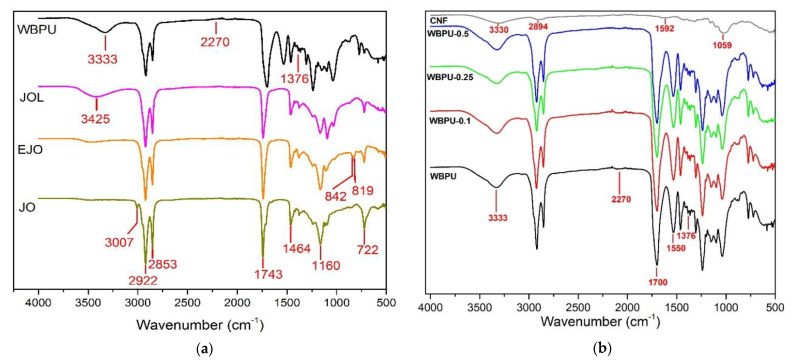
FTIR Spectra of: (**a**) JO, EJO, JOL, WBPU; (**b**) WBPU-CNF and CNF nanocomposite films.

FTIR analysis was monitored for waterborne polyurethane synthesis, as shown in Figure 2. The absorption bands at 3007 cm^−1^, corresponding to the alkene double bond of JO, disappeared upon epoxidation. In the meantime, a doublet band associated with the epoxy group (C-O-C) in the EJO was observed at 842 and 819 cm^−1^, indicating that the Jatropha oil was successfully converted to epoxidized Jatropha oil. During the oxirane ring-opening, the doublet bands of the epoxy group disappeared and new absorption bands at 3425 cm^−1^, corresponding to the stretching vibration of the OH group, were formed, indicating that JOL was produced [5,26]. No absorption band of the OH group vibration was found in the WBPU spectra, indicating that all isocyanate was completely reacted to WBPU production by the OH group of JOL, 1,6 HDO, and DMPA [22]. This was supported by the failure to detect the vibrational band of isocyanate groups (NCOs) of IPDI at 2270 cm^−1^. This also meant that all the residues of the -NCO group were consumed and disappeared after the chain extension reaction. Meanwhile, the FTIR spectrum was detected as shown in Figure 2b. CNF spectrum shows typical bands associated with stretching vibration of hydroxyl, methyl, carboxyl, and pyranose ring ether group were detected, as summarized in Table 2 [22,27]. The influence of CNF on the FTIR spectrum of nanocomposite films is further discussed later in this section.

In the case of neat WBPU film, the characteristic vibrational bands such as N-H stretching (ʋ_N-H_, 3600–3000 cm^–1^), H bonded urethane C=O stretching (ʋ_C=O_, H-bond urethane, 1700 cm^–1^), C-N stretching/N-H bending (ʋ_N-H_/δ_N-H_, 1550 cm^–1^) and symmetric C-H bending (δ_C-H,sym_, 1376 cm^–1^) were identified as listed in Table 2 [22,28]. There was a minimal side reaction to the formation of urea as no observable peak of the carbonyl group (C=O) between 1645 and 1635 cm^–1^.

In the case of WBPU-CNF nanocomposite film with different CNF contents of 0.1 to 0.5 wt.%, all WBPU characteristic bands as polymer matrix have been detected. Characteristic bands associated with N-H stretching (3324 cm^–1^) have been found to shift to a higher wavenumber by adding CNF to WBPU films, as tabulated in Table 3 [19,29,30]. The change in position with increasing CNF content shows the presence of interaction between CNF and the hard segments of WBPU. However, this shift in wavenumber was not significant, and it can therefore be concluded that CNF and the hard segments of WBPU were physically bonded. This is supported by the lack of observation of the H-bonded urethane peak [19,29,30]. A similar finding was made by Cao et al., 2013, who reported that the N-H band of WBPU had moved from 3363 to 3335 cm^-1^, with an addition of 30 wt.% of CNF [29]. In the meantime, during film formation, CNF was embedded in the WBPU matrix, thus preventing the CNF OH group from being visualized by FTIR-ATR as a surface physicochemical analysis leading to a less observable N-H stretching peak [31].

### 3.2. Morphology of WBPU and WBPU-CNF Nanocomposite Films

**Figure 3:**
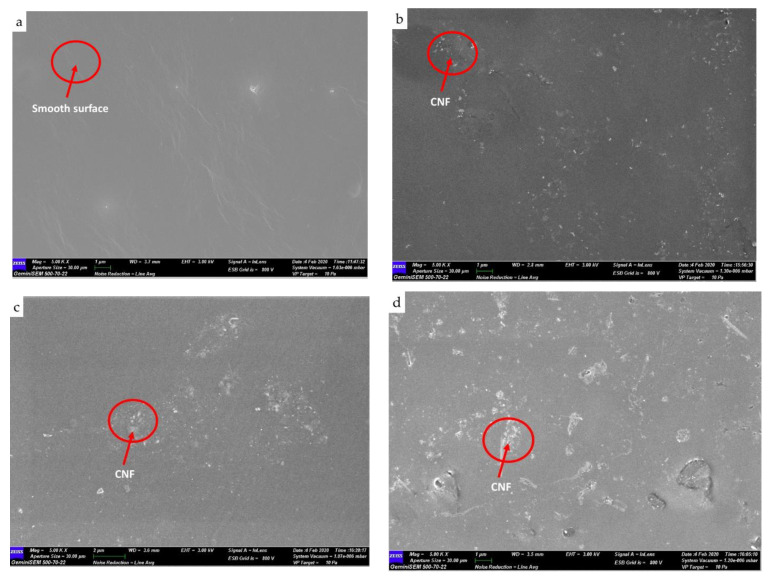
FESEM images of: (**a**) neat WBPU; (**b**) WBPU-0.1; (**c**) WBPU-0.25; (**d**) WBPU-0.5.

Figure 3 shows the images of the FESEM morphology of the WBPU and WBPU-CNF nanocomposite films. The surface of the WBPU-CNF nanocomposite film became rougher compared to the neat WBPU film which is smooth, neat, and clean (Figure 3a) as the loading of CNF increased from 0.1 to 0.5 wt.% (Figure 3b–d). The white dots shown on the FESEM images of the WBPU-CNF nanocomposite are CNF nanoparticles. Lower loadings of CNF (0.1 wt.% and 0.25 wt.%) content have made the WBPU matrix display poor in terms of CNF distribution (Figure 3b,c). The WBPU-0.5 image (Figure 3d) shows a good distribution of CNF in the WBPU matrix. This is supported by white dots with a small size evenly dispersed across the WBPU matrix. This demonstrated the presence of interactions between CNF and the hard segment of the WBPU. Such an event and the homogenous distribution of the CNF in the matrix play an important role in improving the mechanical properties of the resulting nanocomposite films as discussed further in Section 3.3. Similar FESEM images have been reported in the literature [29,32,33,34]. Based on our previous studies, cellulose loadings of more than 1 wt.% show agglomeration, so that its mechanical properties have not been improved [24,34,35]. It is expected that 0.5 wt.% is the optimum ratio for a good homogeneous distribution of CNF in the Jatropha oil-based waterborne polyurethane matrix.

### 3.3. Mechanical Properties of WBPU and WBPU-CNF Nanocomposite Films

**Figure 4:**
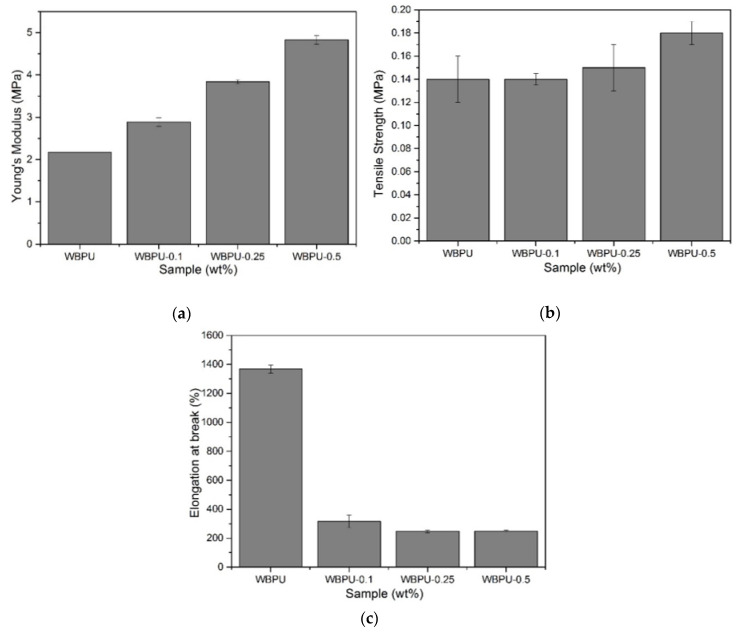
(**a**) Young’s Modulus, (**b**) tensile strength, and (**c**) elongation at break of WBPU and WBPU-CNF nanocomposite films.

WBPU and WBPU-CNF were tested using a universal room temperature testing machine and the results for their mechanical properties are shown in Figure 4. As expected, Young’s modulus and strength value increased and elongation at break decreased as the CNF increased in the nanocomposite series. Young’s modulus increased by up to ~122%. This was due to the CNF loading being below the percolation threshold which showed no excess loading of the filler. The tensile strength values of the composite film increased by up to ~28%. This was due to the more interconnected cellulosic network formed by increasing the content of nanoparticles. The network formed was facilitated by the flexibility of the CNF due to its high aspect ratio and the presence of amorphous domains along nanofibers [32]. These results are consistent with PU composites reinforced by conventional fillers [24,27,36,37,38].

In the case of nanocomposite films, the elongation at break decreased as the CNF loading increased. As CNF loading increased, the nanocomposite film approached the percolation threshold (agglomeration), lowered the area of physical interaction, and resulted in a stress deficiency transfer. This area of heterogeneous and agglomeration has become a stress concentrator and a failure point for nanomaterials [39]. Gao et al. [37] also reported a similar observation. In the case of neat WBPU, the maximum elongation at break value was 1367%. This was due to the presence of a chain extender as well as an amorphous cellulose domain. The literature reported by Saalah et al. [6] shows that Jatropha oil-based waterborne polyurethane obtained 326.2% elongation at break value by using 2-hydroxyethyl methacrylate (HEMA) as an end-capping agent instead of a chain extender. When extended with a chain extender, an increase in elongation at a break of as much as 321% was observed. The present study shows that a higher elongation at break value was achieved with 0.5 wt.% CNF (1367%) compared to soybean oil-based polyurethane reinforced with 0.5 wt.% nanofiller (40%) and poly(Ɛ-caprolactone) based polyurethane reinforced with 1 wt.% nanofiller (12%) [32,39]. There was an increase of as much as 521% and 1973.17% compared to the present study. As a result, the present study shows that the highest elongation at break value can be achieved with only 0.5 wt.% as the optimum filler loading.

**Figure 5:**
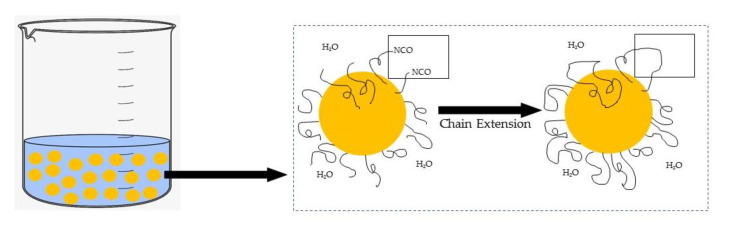
The reaction of chain extension.

On the other hand, and even more important in this study, the contribution of the chain extender to the improvement of the stretchability properties should be highlighted. The elastomeric properties increased when the chain extender used did not show urea functional groups. Therefore, a chain extender containing a hydroxyl group is preferable. Based on the work carried out by Delpech & Coutinho [40], PU extended with ethylenediamine (EDA) and hydrazine (HYD) as a chain extender has the lowest elastomer character and therefore the highest rigidity compared to ethylene glycol (EG). EDA and HYD containing-NH_2_ groups formed poly(urethane-urea)s, resulting in more rigid films due to urea bonding, which increases the hydrogen bonding density compared to EG. Following the same behavior discussed above, the presence of a chain extender helped to increase the length of the hard segment, resulting in an elongation of 1370% when the WBPU film breaks. The extension step of the chain is illustrated in Figure 5.

### 3.4. Thermal Properties of WBPU and WBPU-CNF Nanocomposite Films

**Figure 6:**
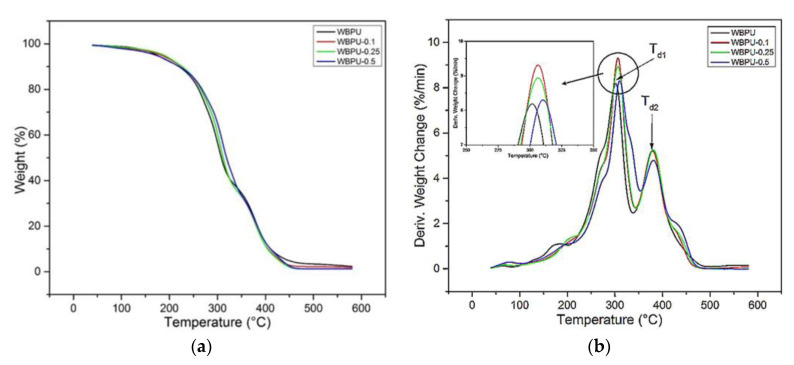
(**a**) TGA and (**b**) DTG curve for WBPU and WBPU-CNF nanocomposite films.

The thermal degradation of composites was studied by thermogravimetric analysis. TG and DTG thermograms of net WBPU and WBPU-CNF are shown in Figure 6a,b, respectively, and the thermal parameters are summarised in Table 4.

In the case of a neat WBPU film, a two-step weight loss at ~299 °C and ~378 °C was detected. The first weight losses at T_d1_ ~299 °C and T_d2_ ~378 °C corresponded to the thermal decomposition of the hard segment (urethane group) and soft segment (JOL component) in the WBPU chains with a weight loss of 53.82% and 40.72%, respectively. This is a common polyurethane thermogram because the soft segment had higher thermal stability than the hard segment [35,41]. The results obtained are consistent with the previous work carried out by others [24,27,42,43]. No thermogram peak was observed at 333 °C, corresponding to the thermal decomposition of the urea group. WBPU was successfully synthesized with minimal side reactions, as supported by FT-IR analysis.

In the case of nanocomposite film with 0.1 to 0.5 wt.% CNF loading, the first and second temperatures of thermal decomposition, *T_d1_* and *T_d2_*, nanocomposite film shifted to higher temperatures as CNF loading increased, as can be seen in Figure 6a,b, and Table 4. The residual composite film at 600 °C decreased from ~0.99 to ~0.0001 wt.% as the CNF content increased from 0.1 to 0.5 wt.%. This improvement in the thermal stability of the nanocomposite film was believed to be due to the mechanical bonding between WBPU and CNF, as discussed in the FTIR analyses referred to above. Similar findings were also reported in other studies [43,44,45]. It was noted that the increase in the thermal decomposition of the soft segment (JOL component) was due to the fact that the hard segment of the WBPU and CNF, which shows the presence of mechanical bonding, was decomposed at a lower temperature (*T_d1_*) to form a char layer on the nanocomposite film, acting as a retardant or barrier to the decomposition of the soft segment at a high temperature [22].

### 3.5. Contact Angle of WBPU and WBPU-CNF Nanocomposite Films

**Figure 7:**
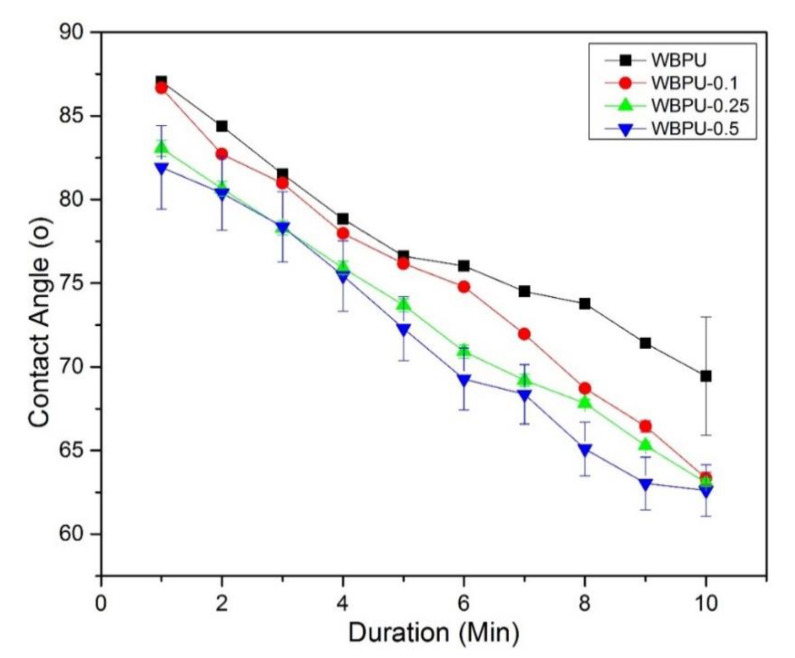
Contact Angle for WBPU and WBPU-CNF nanocomposite films.

The hydrophobicity/hydrophilicity of the film surfaces was observed by measuring the static contact angle as shown in Figure 7.

The water droplets of the neat WBPU have hardly changed on the surface of the neat film during the test process, indicating a small absorption of the film water. In the case of the WBPU-CNF composite, the contact angle decreased gradually as the CNF content increased from 0.1 to 0.5 wt.%. The reason could be attributed to the hydrophilic nature of the CNF [45], while the higher surface roughness and lower surface free energy have affected the hydrophobic surface of the film [46]. It could be seen in the FESEM images that WBPU-0.5 showed a bit of surface roughness that supported this finding because the WBPU-0.5 nanocomposite film indicated the lowest angle of contact with water. In the present study, the contact angle of the neat WBPU and WBPU-CNF nanocomposite films was between 79° and 87°, which was almost hydrophobic (90°). This was attributed to the molecular level movement restriction further explained in Section 3.6.

### 3.6. Water Uptake of WBPU and WBPU-CNF Nanocomposite Films

**Figure 8:**
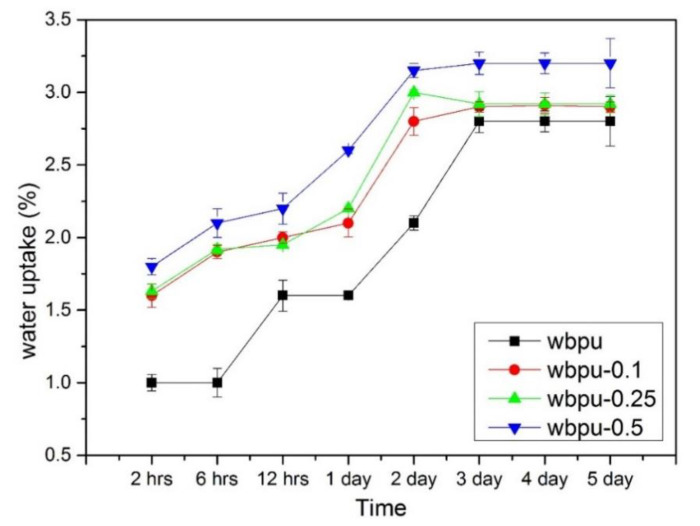
Water uptake of WBPU and WBPU-CNF nanocomposite films.

The water uptake as a function of time for WBPU and WBPU-CNF nanocomposite films is shown in Figure 8.

The water uptake for all WBPU and WBPU-CNF nanocomposite films increased with time and plateaued at three days and above. The water uptake of WBPU-0.5 was the highest and neat WBPU was the lowest. Due to the natural hydrophobic properties of the PU, the lowest water uptake of neat WBPU was obtained. This high hydrophobic value could be attributed to the high hard segment content that was responsible for the high crosslinking density of hydrogen bonding between the segmental PU [47]. In the case of WBPU-CNF nanocomposite films, the increasing trend of water uptake from WBPU-0.1 to WBPU-0.5 was due to the hydrophilic nature of CNF. However, this increase did not show any significant difference as the CNF was covered with the PU matrix as discussed in Section 3.2. Meanwhile, the lack of presence of a side reaction (urea) in neat WBPU and WBPU-CNF nanocomposites films also contributed to this lower water uptake. Furthermore, the complex structure of the soft and hard segments formed as a result of the decrease of the absorbed water and restricted the movement of the molecular chain. In addition, the complex structure of the soft and hard segments formed as a result of the decrease in absorbed water and restricted the movement of the molecular chain during the swelling process, leading to a reduction in mechanical properties [48]. It can be concluded that the water uptake of neat WBPU and its composites films was very low and did not exceed 3.5%. This indicates that hydrophobic behavior is still dominant in WBPU-CNF films. This was supported by the result obtained from the contact angle of 79° to 87°, which was almost hydrophobic. Water uptake properties are important for coatings, especially for corrosion applications [49,50,51]. The high water absorption behavior will affect the efficiency of the coating materials, which are mostly polyurethane-based.

## 4. Conclusions

In this work, Jatropha oil-based, water-borne polyurethane (WBPU) nanocomposite film with cellulose nanofibres (CNF) was prepared and characterized. We determined the effect of CNF filler on the mechanical and thermal properties of the nanocomposite film. A significant effect on mechanical properties was observed over a range of 0.1 to 0.5 wt% CNF loading. This could be attributed to the well dispersed CNF within the polymer matrix, as confirmed with FESEM images. Young’s modulus and tensile strength of the composite films were increased by 55% and 22%, respectively, compared to the neat WBPU. Young’s modulus and tensile strength were increased by 55% and 22%, respectively, after being incorporated into CNF. With the presence of a chain extender, the neat WBPU achieved 1370% elongation at break and reduced stretchability as the content of CNF increases. CNF loading strongly interacts with the hard segment of the WBPU matrix shifting the hard segment thermal degradation to higher temperature with increasing CNF loads. The contact angle of WBPU-CNF nanocomposite films is reduced compared to the neat WBPU. However, the water absorption activities of neat WBPU and WBPU-CNF nanocomposite films show low water absorption values that do not exceed 3.5%.

## Figures and Tables

**Table 1:** Formulation of WBPU synthesis.

	CNF ^1^	JOL ^1^	DMPA ^1^	IPDI ^1^	HDO ^1^	TEA ^1^	Water ^1^
WBPU	0	52.54	11.2	24.52	5.17	6.57	16
WBPU-0.1	0.1	52.54	11.2	24.52	5.17	6.57	16
WBPU-0.25	0.25	52.54	11.2	24.52	5.17	6.57	16
WBPU-0.5	0.5	52.54	11.2	24.52	5.17	6.57	16

^1^ Unit is in weight percentage.

**Table 2:** Characterization of vibrational bands of WBPU.

Wavenumber (cm^−1^)	Band Assignment of Neat WBPU	Wavenumber (cm^−1^)	Band Assignment of CNF
3600–3000	${ʋ}_{N-H}$	~3372	$\nu_{O-H}$
~1700	${ʋ}_{C=O,\;\;\;H-bond\;urethane,\;\;\;}$	~2894	$\nu_{C-H}$
~1550	${ʋ}_{N-H}/\delta_{N-H}$	~1592	$\nu_{C-O}$
~1376	$\delta_{C-H,\;sym}$	~1059	$\nu_{C-O-C}$

**Table 3:** Characteristic vibrational band influence by addition of CNF.

Samples	${ʋ}_{N-H}$ (cm ^−1^)	${ʋ}_{C=O,\;\;\;H-bond\;urethane,\;}$ (cm ^−1^)
WBPU	3333	1700
WBPU-0.1	3326	1700
WBPU-0.25	3326	1700
WBPU-0.5	3321	1700

**Table 4:** Thermal Properties of WBPU and WBPU-CNF nanocomposite films.

	*T_dec_* (°C)		Weight Loss (%)		Residue at 600 °C (%)
	*T_d1_*	*T_d2_*	*W_d1_*	*W_d2_*	
WBPU	299.76	378.79	53.82	40.72	0.9962
WBPU-0.1	305.61	378.30	57.74	37.66	0.4544
WBPU-0.25	306.10	380.25	58.30	38.56	0.0113
WBPU-0.5	313.42	380.25	60.70	35.25	0.0001

## Data Availability

The data presented in this study are available on request from the corresponding author.
